# Treatment with (R)-α-methylhistamine or IL4 stimulates mucin production and decreases *Helicobacter pylori* density in the murine stomach

**DOI:** 10.1080/21505594.2025.2530173

**Published:** 2025-07-16

**Authors:** Licínia Santos, Sinan Sharba, John Benktander, Stefany Ojaimi Loibman, Macarena P. Quintana-Hayashi, Mattias Erhardsson, Sara K. Lindén

**Affiliations:** Department of Medical Biochemistry and Cell Biology, Institute of Biomedicine, Sahlgrenska Academy, University of Gothenburg, Gothenburg, Sweden

**Keywords:** *H. pylori*, mucin, mucus, stomach, mouse

## Abstract

*Helicobacter pylori* is the most common gastric pathogen. *H. pylori* is prone to develop antibiotic resistance and recurrence after therapy makes treatment problematic. *H. pylori* can be detected attached to the gastric epithelial cells; however, it is mostly found within the gastric mucus. *Helicobacter* species infections impair the mucus barrier by decreasing the binding ability of the mucins, decreasing the growth-limiting activity of mucins and decreasing mucin production. The current study aimed to restore mucin production in the male C57BL/6 mouse *H. pylori* (SS1) infection model and evaluate its effects on *H. pylori* density. Mice infected with SS1 were treated with (R)-α-methylhistamine (RαMH) or interleukin-4 (IL4). Treatment with RαMH or IL4 restored mucin production and decreased gastric *H. pylori* density compared to mock-treated infected mice. Treatment with RαMH and IL4 did not affect serum anti-*H. pylori* IgG levels, expression of antimicrobial peptides or *H. pylori* virulence factors. Further, RαMH did not have cytotoxic effects on *H. pylori*. However, the expression of cytokines (*Tnf* and *Il4)*, factors related to mucus production (*Tff1*, *Spedf, Stat6,* and *Ptgs1*), and mucin O-glycan sialylation levels differed between mice treated with RαMH and IL4. This suggests that increased mucus production can have similar effects on pathogen density in spite of differences in the local niche. In conclusion, agents that stimulate mucin production in the gastric mucosa have the potential to aid in the removal of pathogens from the gastric niche.

## Graphical abstract

**Figure:**
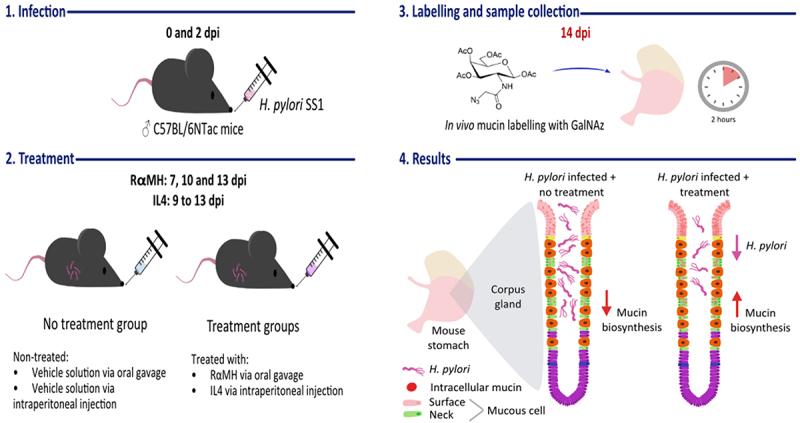

## Introduction

*Helicobacter pylori*, a gram-negative rod-shaped microaerophilic bacterium, has several well-described virulence factors (including *ureA, flaA, cagA, vacA*) that contribute to its success in colonizing the gastric mucosa of half of the world’s population [1–4]. *H. pylori* infection seems to mainly occur at an early age and once acquired results in long-term inflammation in the gastric mucosa. Despite being asymptomatic at times, infected individuals may develop non-ulcer dyspepsia, peptic ulcer disease, mucosa-associated lymphoid tissue lymphoma, and gastric adenocarcinoma (the fifth most common cause of death due to malignancy) [5,6]. *H. pylori* has been classified as a class 1 carcinogen by the International Agency for Research on Cancer [7].

To treat the infection, a combination of antibiotics and proton pump inhibitors are commonly used, however, increasing development of antibiotic resistance makes the treatment problematic [8].

In the stomach, *H. pylori* can be found attached to the gastric epithelial cells but the majority reside within the mucus [9]. Mucus is a viscous adherent secretion synthesized by the gastric mucous cells in the columnar epithelium that protects the epithelium against harmful agents. The mucosal surfaces are the most common routes used by pathogens to enter the body, thus the mucus layer is the first barrier pathogens encounter [10,11]. The mucus layer is comprised by large glycoproteins named mucins that form a scaffold keeping other defensive molecules secreted by the epithelial cells (e.g. antimicrobial peptides [AMPs]) in a strategic location [12]. To successfully colonize the host, *Helicobacter* species impair the mucus barrier by decreasing the binding ability of the mucins [13,14], decreasing the growth-limiting activity of mucins [14] and decreasing the mucus production [15]. Mucins can bind and remove several pathogens (including *H. pylori*) and regulate their growth and virulence [10,16–18]. Mice lacking the Muc1 and Muc5AC mucins have a higher *H. pylori* density after infection than wild-type mice [19,20]. Furthermore, rhesus monkeys with mucins that bind *H. pylori* more effectively have a lower *H. pylori* density in their stomachs than monkeys with low *H. pylori* binding ability [13]. Together with that MUC1 can act as a steric hinder and as a releasable decoy for *H. pylori* binding to the epithelial cell surface [20], these findings suggest that mucin binding to *H. pylori* contributes to removing the bacteria from the gastric niche. Gastric mucus production decreases in *H. pylori*-infected mice [15] and in the murine colon during mid-point *Citrobacter rodentium* infection [21]. However, during *C. rodentium* clearance, the mucus production increases, concomitant with pathogen displacement from the mucosal surface [21,22]. By treating mice with Interleukin 4 (IL4), which enhances colonic mucus production, the removal of *C. rodentium* from the epithelial surface is induced at an earlier time point than in mock-treated mice [21].

Different compounds, such as (R)-α-methylhistamine (RαMH) and IL4, have been indicated to influence mucin production [21,23]. RαMH is a selective agonist of the histamine-3 (H_3_) receptor [24]. In rats, RαMH enhances the number and volume of gastric mucous cells as detected by Alcian Blue Periodic Acid Schiff (AB/PAS) staining and appears to increase the thickness of the secreted mucus layer [23]. Furthermore, immunohistochemical studies with a thymidine analog showed that RαMH induces the proliferation of rat gastric epithelial cells [25,26]. Histamine-3 agonists, such as RαMH, are indicated to be involved in maintaining gastric mucosal integrity by modulating prostaglandin E_2_ (PGE_2_) production [27]. IL4 is produced by T cells, mast cells, and basophils and has many biological roles in innate and adaptive immunity. Moreover, IL4 contributes to increased antibody responses during infection [28]. *H. pylori* eradication has been shown to increase IL4 levels [29] and IL4 treatment decreases pathogen density and the severity of gastritis in *H. felis*-infected mice [30]. In addition, IL4 increases mucus production in colonic cells, especially when in combination with bacterial infection [21]. Furthermore, IL4 increases AB/PAS positive staining in a human pulmonary mucoepidermoid carcinoma cell line as well as in the mouse airway, indicating increased mucus production [31].

Based on the above, we hypothesized that RαMH and IL4 would restore the mucin inhibition caused by *H. pylori* infection and decrease the bacterial density in the murine stomach.

## Methods

### Ethics

The Göteborgs Djurförsöksetiska Nämnd (Ethic No. 52–2021) approved all experimental procedures based on the regulation from Djurskyddsförordningen DFS 2004:4.

### Animals

Six-week-old, specific-pathogen-free, wild-type male black C57BL/6Ntac mice (Taconic Biosciences, Denmark) were divided into cages by the vendor and housed in individually ventilated cages in the same room at the core facility for Experimental Biomedicine (EBM), University of Gothenburg. The animals had a 12-h light/dark cycle and access to water and food *ad libitum*. The mice were quarantined in the animal facilities for a week before starting the experiment. The infections, treatments, and sample collection were performed at the same location.

### Infection

The mouse-adapted *H. pylori* SS1, stored at −80°C in Brain-Heart infusion (BHI) broth (CM1135, Oxoid) with 20% glycerol, was used as stock culture. The bacteria were cultured for 4 days on Brucella agar (CM0169, Oxoid) supplemented with 10% horse blood in microaerobic conditions. Then, the bacteria were cultured overnight in BHI supplemented with 10% fetal bovine serum (FBS) under microaerobic conditions. For infection, a suspension of bacteria in phosphate-buffered solution (PBS) with an adjusted optical density at 600 nm (OD_600_) of 1.0 was prepared. The bacterial suspension was visually inspected under the microscope to ensure they were healthy and viable (>90% motile, helical, and not coccoid). To confirm that the mice were infected with the expected dose, serial dilutions of the suspension prepared for infection were plated on Brucella blood agar plates and the colonies formed were counted after 4 days of incubation. For infection, the mice received two doses, with a day interval, of 200 µl of *H. pylori* SS1 (3.5 × 10^7^ CFU/mouse) suspended in PBS by oral gavage using a feeding needle. The non-infected controls received vehicle solution (200 µl of PBS).

### Treatments

The mice were divided into groups and received treatments or vehicle solutions accordingly.

RαMH: the group of infected mice treated with RαMH were treated with 100 mg/kg RαMH (J61636.MC, Alfa Aesar) in 200 µl PBS containing 6.25% dimethylsulfoxide (DMSO). The group of non-infected controls and the group of *H. pylori*-infected vehicle-treated mice received 200 µl PBS containing 6.25% DMSO (vehicle solution). The treatment and vehicle were administered by oral gavage with a feeding needle at 7-, 10-, and 13 days post-infection (dpi). The group of non-infected control mice and the group of *H. pylori*-infected vehicle-treated mice were handled as the group treated with RαMH but only received vehicle solutions.

IL4: *H. pylori*-infected mice treated with IL4 were given 0.2 µg/mouse of IL4 (SRP3211, Sigma-Aldrich) in PBS. The intraperitoneal injections (100 µl) were administrated daily) from day 10 to 13 pi. Non-infected controls and *H. pylori*-infected vehicle-treated mice received intraperitoneal injections of 100 µl PBS instead on the same days.

### *In vivo* mucin labeling and sample collection

GalNAz incorporates into the core region of mucin O-glycans and has been used to analyze mucin production and transport in cell lines [32], mice [15] and fish [33,34]. A total volume of 300 µl per mouse was prepared by dissolving 2.6 mg of GalNAz (900915, Sigma-Aldrich) in 50 µl DMSO followed by dilution in PBS. The GalNAz injections were given intraperitoneally at 14 dpi, and mice were sacrificed 2 h after the injection by cervical dislocation under anesthesia with isoflurane. Whole blood samples from each mouse were collected upon sacrifice. Then, the stomachs were harvested and opened along the greater curvature followed by a gentle wash in sterile PBS to remove chyme. A stripe of the lesser curvature of the stomach containing forestomach, corpus, and antrum was fixed in buffered formaldehyde 4% aqueous solution (pH 7.0 ± 0.2, 9713.100, VWR) for 24 h. The fixed tissue was paraffin-embedded and histological slides were prepared with 4-µm-thick tissue ribbons. Additionally, two pieces of the corpus (approximately 4 × 4 mm) were collected. One was placed in RNA-later (R0901, Sigma-Aldrich) and stored at 4°C; the other one was immediately frozen in dry ice. Both samples were later stored at −80°C until use.

### Histology

To assess gastritis, Hematoxylin and Eosin (H&E) staining was performed by dipping deparaffinized and hydrated slides in hematoxylin and after washing in water, in eosin. The slides were washed once more and dehydrated with increasing concentrations of ethanol, dipped in isopropanol followed by xylene, and finally mounted. Gastritis was scored in a blinded fashion following the criteria described in [35] at 40x magnification.

For fluorescent detection of GalNAz, tissue samples were incubated with 30 µl of the reaction mix from the tetramethylrhodamine (TAMRA) glycoprotein detection kit (C33370, Thermo Fisher Scientific) and the following steps were performed as described elsewhere [21]. The localization of incorporated GalNAz was evaluated in a blinded fashion at 40x magnification on an Eclipse 90i fluorescence microscope (Nikon), by assessing 10 mucous cells located on the corpus surface of each mouse stomach. The cells were divided into four regions (perinuclear, mid-cytoplasm, near the apical cell surface and at the apical cell surface) and each region received a score from 0 to 4 based on the amount of TAMRA fluorescence in that region.

Bacteria were visualized with fluorescent *in situ* hybridization (FISH) following the protocol as previously described [36]. The probes used were as follows: EUB338 labeled with Cy3.5 (5’GCTGCCTCCCGTAGGAGT3’) to detect all bacteria and *Helicobacter* labeled with Alexa 488 (5’ TCTCAGGCCGGATACCCGTCATAGCCT3’) to identify only *Helicobacter* species. The *Helicobacter* in the tissue were counted in the corpus pits as this area is less susceptible to artifacts due to loss of mucus compared to the gastric surface. *Helicobacter* were counted in five fields of view with a 40x magnification on an Eclipse 90i fluorescence microscope (Nikon) in a blinded fashion. The results are presented as the average count of the five fields.

### Glycan analysis with liquid chromatography-mass spectrometry (LC-MS)

A crude mucin extraction was made from murine corpus, analyzed using liquid chromatography-electrospray ionization tandem mass spectrometry (LC/MS) and LC/MS and MS^2^ spectra interpretation were performed as described previously [37]. Using RStudio (2024.12.1 build 563) with the package ropls (1.34.0) [38], an Orthogonal Partial Least Square Discriminant Analysis (OPLS-DA) [39] was performed on the O-glycan MS results to find the glycans best-predicting differences between groups. From the top 10 variable influence on projection (VIP), the glycans with a relative abundance >0.5% were analysed using the Kruskal–Wallis H-test.

### Evaluation of anti-*H. pylori* IgG levels in murine serum

Murine sera obtained by centrifugation of the blood at 3000 *g* for 10 min at 4°C was used to determine the antibody response to *H. pylori* at 14 dpi by Enzyme-Linked Immunosorbent Assay (ELISA). *H. pylori* SS1 was grown as mentioned above, resuspended in sterile PBS, and sonicated (Sonics, VibraCell) six times for 2 min (amplitude 60, pulse 2.0) with 2 min intervals between sonication. The suspension was centrifugated at 13 900 *g* for 30 min at 4°C. The resulting pellet (membrane protein) was resuspended in sterile PBS and the protein concentration was quantified using the Pierce BCA Protein Assay Kit (23227, Thermo Scientific) following the manufacturer’s protocol. MaxiSorp 96-well plates (430341, Thermo Scientific) were coated with 20 µg/ml of membrane protein overnight at 4°C and the assay was performed as previously described [21]. Serum from an *H. pylori*-infected mouse 14-weeks pi was used as a positive control (PC) and serum from a germ-free mouse as a negative control (NC). The absorbance was measured at 450 nm on CLARIOstar Plus microplate reader (BMG Labtech).

### Gene expression analysis

The murine corpus was homogenized in lysis buffer with a tissue rotor (IKA T10 basic – ultra turrax) and total RNA (bacterial and murine) was extracted with RNeasy Mini Kit (74104, Qiagen) with on-column DNase digestion according to the manufacturer’s recommendations. The concentration and quality of the extracted RNA were measured with NanoDrop (Thermo Fisher Scientific). The cDNA synthesis was done with Quantitect Reverse Transcription Kit (205310, Qiagen) on 1000 ng RNA. Real-time polymerase chain reaction (RT-PCR) was performed in duplicates in hard-shell PCR plates, 96-well thin wall (HSP9665, BioRad Laboratories) on a CFX96 Real-Time System (BioRad Laboratories) following the protocol: 95°C for 2 min, then 40 cycles consisting of denaturation for 5 s at 95°C followed by 30 s at 60°C (annealing and extension steps).

The murine gene expression was analyzed using 100 ng of cDNA, 20x PrimePCR SBR Green assay (BioRad; primers are listed in Table 1) and 2x SsoAdvanced Universal SYBR Green SuperMix (1725271, Bio-Rad Laboratories) following the manufacturer’s recommendations. The β actin (*Actb*) was used as the reference gene. The results were normalized to the mean gene expression of non-infected control mice.

**Table 1.** Primers used for gene expression analysis.

Gene	Unique assay ID (Bio-Rad)
*Actb*	qMmuCED0027505
*Defb1*	qMmuCID0008786
*Ifng*	qMmuCID0006268
*Il4*	qMmuCED0044969
*Ltf*	qMmuCID0022149
*Myd88*	*qMmuCID0015570*
*Nfkb1*	*qMmuCED0047222*
*Ptgs1*	*qMmuCED0050070*
*Ptgs2*	*qMmuCED0003742*
*Slpi*	*qMmuCED0004965*
*Spdef*	*qMmuCED0046601*
*Stat6*	*qMmuCID0006404*
*Tff1*	*qMmuCED0047222*
*Tnf*	*qMmuCED0004141*

*H. pylori* virulence factors were analyzed using 100 ng of cDNA, and primers at a final concentration of 5 mM. Each reaction had a final volume of 10 µl containing 5 µl of 2x iTAQ Universal SYBR Green SuperMix (1725121, BioRad Laboratories). The *H. pylori* SS1 primers were designed with NCBI primer blast (Table 2). *Helicobacter* 16s was used as the reference gene. The results were normalized to the mean gene expression of *H. pylori*-infected vehicle-treated mice.

**Table 2.** H. pylori SS1 primers.

Gene	Forward Primer	Reverse Primer
*16s*	GGAGTACGGTCGCAAGATTAAA	CTAGCGGATTCTCTCAATGTCAA
*ureA (HPYLSS1_00068)*	GCTGGTAAAAAGACTGCGGC	CACGGTTACGAGTTTGGTCC
*cagA (HPYLSS1_00752)*	CACCACCCACATACAAGGCT	GTCAGCGACTCCCTCAACAT
*flaA (HPYLSS1_00808)*	CGGGCAAGCGTTATTGTCTG	GCGATACGAACCTGACCGAT
*vacA (HPYLSS1_00674)*	GGAAAGCCAGCTCTACGGTT	GTATTGCAAACGGCCCATCC

The Ct values of the β actin (mean: 18.87) for the control mice indicated that a sufficient amount of total host mRNA was present for the analysis; however, the Ct values of some analyzed genes (mean Ct: *Tnf* 35.4, *Ifng*: 36.4, *Il4* 32.9, *Defb1* 30.6, *Ltf* 31.2, *Slpi* 27.0, *Myd88* 26.94, *Nfkb1* 26.91, *Spdef* 26.63, *Stat6* 26.71, *Tff1* 13.54, *Ptgs1* 24.96, and *Ptgs2* 32.09) were high; thus, the amount of template for *Tnf* and *Ifng* was low. No amplification was detected in the negative control samples (water) for any gene. Since *H. pylori* mRNA is in low abundance compared to the host mRNA, the Ct values of the *H. pylori* housekeeping gene *16s* were higher (mean: 31.4) than for β actin. The mean Ct values of the virulence factors studied were as follows: *ureA* 32.7, *flaA* 33.8, cagA 34.7, and vacA 35.3. No amplification of 1*6s, ureA, flaA, cagA*, or *vacA* was detected in non-infected control mice or the assay’s negative control.

### *H. pylori* viability assay

*H. pylori* SS1 were cultured in Brucella Blood agar for 4 days, then harvested and resuspended in Brucella broth (OD_600_ = 0.1). To assess viability, the *H. pylori* suspension was mixed with RealTime-Glo™ MT Cell Viability Assay (G9711, Promega), following the manufacturer’s recommendations, and 170 µl of this suspension was added to wells in a white opaque 96-well plate (353296, Falcon). Subsequently, 30 µl of vehicle solution (PBS with 6.25% DMSO) or RαMH was added. The experiment was carried out at two concentrations: (1) 12.5 mg/ml RαMH in PBS containing 6.25% DMSO, i.e. the same concentration as used in the *in vivo* experiments, and (2) the reagents diluted further 2.5 times in PBS. The plates were incubated at 37°C with 5% CO_2_ and 10% O_2_ for 4 h in a CLARIOstar Plus (BMG Labtech) microplate reader. The assay measured the substrate-reducing potential of viable cells (luminescence).

### Randomization

Infection and treatments were performed in no defined order. *In vivo* labeling and sample collection were performed sequentially starting with non-infected controls followed by *H. pylori-infected* vehicle-treated mice and lastly the treatment groups.

### Statistical analysis

**Figure 1.**
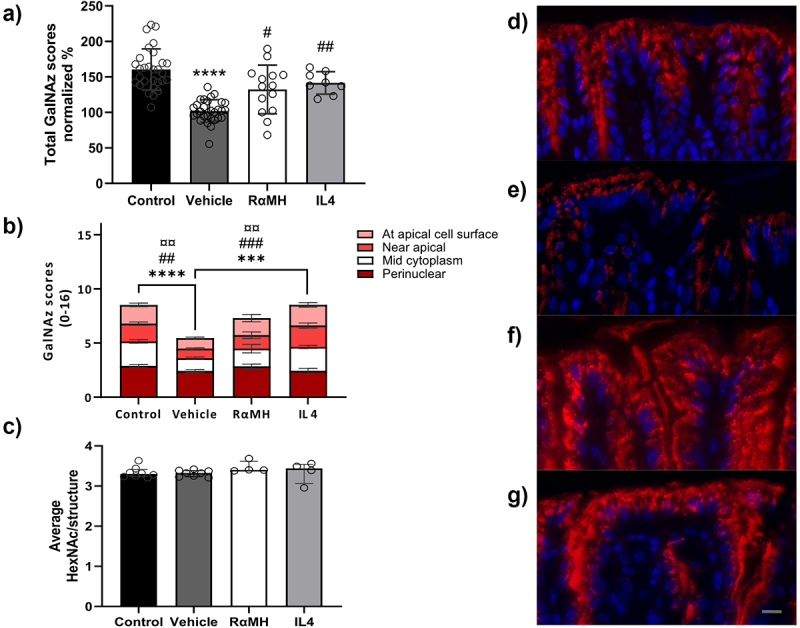
Mucin production and transport through murine gastric mucus-producing cells in *H. pylori-*infected mice treated with RαMH and IL4. Non-infected control mice (Control), *H. pylori-*infected vehicle-treated mice (Vehicle), *H. pylori*-infected mice treated with RαMH (RαMH) and *H. pylori*-infected mice treated with IL4 (IL4) were injected with the metabolic label GalNAz and harvested after two hours. (a) Blinded visual semi-quantification of the intensity of incorporated GalNAz (detected by TAMRA) in mucous cells of control (*n* = 19), vehicle (*n* = 29), RαMH (*n* = 14) and IL4 (*n* = 8) mice. Each location received a score of 0–4 based on intensity and this graph shows the sum of all scores for each group. Each circle represents the score for one mouse. The results were calculated into percentages compared to non-infected vehicle-treated mice to allow for pooling results from five experiments into the same graph. ****p < 0.0001 compared to non-infected controls; # *p* > 0.05, ## *p* > 0.01 compared to *H. pylori-*infected vehicle-treated; Kruskal–Wallis test by ranks followed by Dunn’s multiple comparison test, mean ± S.E.M. (b) An example of an individual experiment visualizing changes in label incorporation in different cellular compartments in surface mucous cell of control (*n* = 11), vehicle (*n* = 11), RαMH (*n* = 5), and IL4 (*n* = 5) mice. ***p < 0.001, ****p < 0.0001 mark the differences in the mid cytoplasm compartment; ## *p* < 0.01, ### *p* < 0.001 mark the differences in the near apical compartment and ¤¤ *p* < 0.01 mark the differences at the apical cell surface; two-way ANOVA, Tukey’s post hoc test, mean ± S.E.M. (c) Average number of HexNAc per O-glycan in mucus from the mouse corpus of control (*n* = 8), vehicle (*n* = 8), RαMH (*n* = 4), and IL4 (*n* = 4) mice. O-glycans were released and analyzed by LC-MS. The HexNAc abundance was analyzed to control for that potential changes in HexNAc content could affect the level of incorporation of metabolic label. No significant differences between the groups were observed; Kruskal–Wallis test by ranks followed by Dunn’s multiple comparisons, bars represent median ± IQR. (d-g) Representative images selected from mice that obtained a median score of incorporated GalNAz in mouse corpus surface cells 2 h after intraperitoneal injection, TAMRA (red, visualize incorporated GalNAz), and DAPI (blue, nucleus), gray scale bar 10 µm. (c) Non-infected control, (d) *H. pylori-*infected vehicle-treated, (e) *H. pylori*-infected treated with RαMH, (f) *H. pylori*-infected treated with IL4. Larger representative images from another two mice from each group are available in supplementary Figure S1.

All tests were performed using Prism (GraphPad Software, version 9.4). The Shapiro–Wilk test was performed to assess normality. The ROUT test with a Q of 1% was used to identify outliers. The Kruskal–Wallis H test (one-way analysis of variance [ANOVA] on ranks) followed by Dunn’s multiple comparisons test was used for non-normally distributed sample groups and values expressed as median ± interquartile range (IQR). The Kruskal–Wallis H test was also used for Figure 1a although the data passed the normality test, since the variables for this figure are not on a continuous scale.

For normally distributed samples, ANOVA with Tukey’s post hoc test and two-way ANOVA were used to compare data from more than two experimental groups. Values were expressed as mean ± S.E.M. The p-values under 0.05 were considered significant.

### Guidelines

This manuscript follows the ARRIVE 2.0 guidelines.

## Results

### RαMH and IL4 restore mucin production in *H.* *pylori*-infected murine stomachs

The mucin production and transport en route secretion in mucous cells were evaluated by metabolic labeling with GalNAz. One hour after injection, it is possible to observe newly synthesized mucins in the peri- and supranuclear compartment of the gastric epithelial cells and during the following couple of hours, the mucins move through the cell toward the cell surface [15]. Therefore, the quantity and location of the mucins were assessed 2-h post-GalNAz injection. Since the mucosal alterations caused by *H. pylori* primarily target the murine corpus, alterations in mucin synthesis were evaluated in the surface mucous cells of the corpus. In line with previously published results [15], mice infected with *H. pylori* had reduced mucin biosynthesis compared to non-infected controls (*p* < 0.0001, Figure 1a,d,e). RαMH and IL4 increased mucin biosynthesis in the *H. pylori*-infected mice (*p* < 0.05 and *p* < 0.01, Figure 1a,e–g). In the RαMH or IL4-treated mice, the mucin biosynthesis level was restored to levels similar to those found in non-infected mice (Figure 1a,d–g and Supplementary Figure S1a-h).

The change in the level of incorporated label was mainly located in the upper half of the cell: at 2-h post-GalNAz injection, the GalNAz label was incorporated in the entire cell, from the perinuclear compartment to the apical cell surface in non-infected mice (Figure 1b,d). In *H. pylori*-infected mice, similar levels of label were incorporated in the perinuclear region of the cell as in non-infected mice, whereas less label was found at the apical half of the mucous cells, i.e. near the apical surface and mid- cytoplasm compartments of the mucous cells (Figure 1b,e, *p* < 0.0001). In mice treated with IL4, the levels of label detected at the apical, near the apical surface and mid-cytoplasm compartments of the mucous cells increased compared to infected vehicle-treated mice (*p* < 0.01), with a similar trend for RαMH (Figure 1b,f–g).

To ensure that differences in incorporated GalNAz label were not due to glycosylation changes, mucin O-glycans from the mouse corpus were analyzed by LC-MS. Indeed, no differences in the relative abundance of HexNAc per structure were observed between the groups (Figure 1c).

### Administration of RαMH reduced histological activity index (HAI) in infected mice with a similar trend for IL4-treated mice

**Figure 2.**
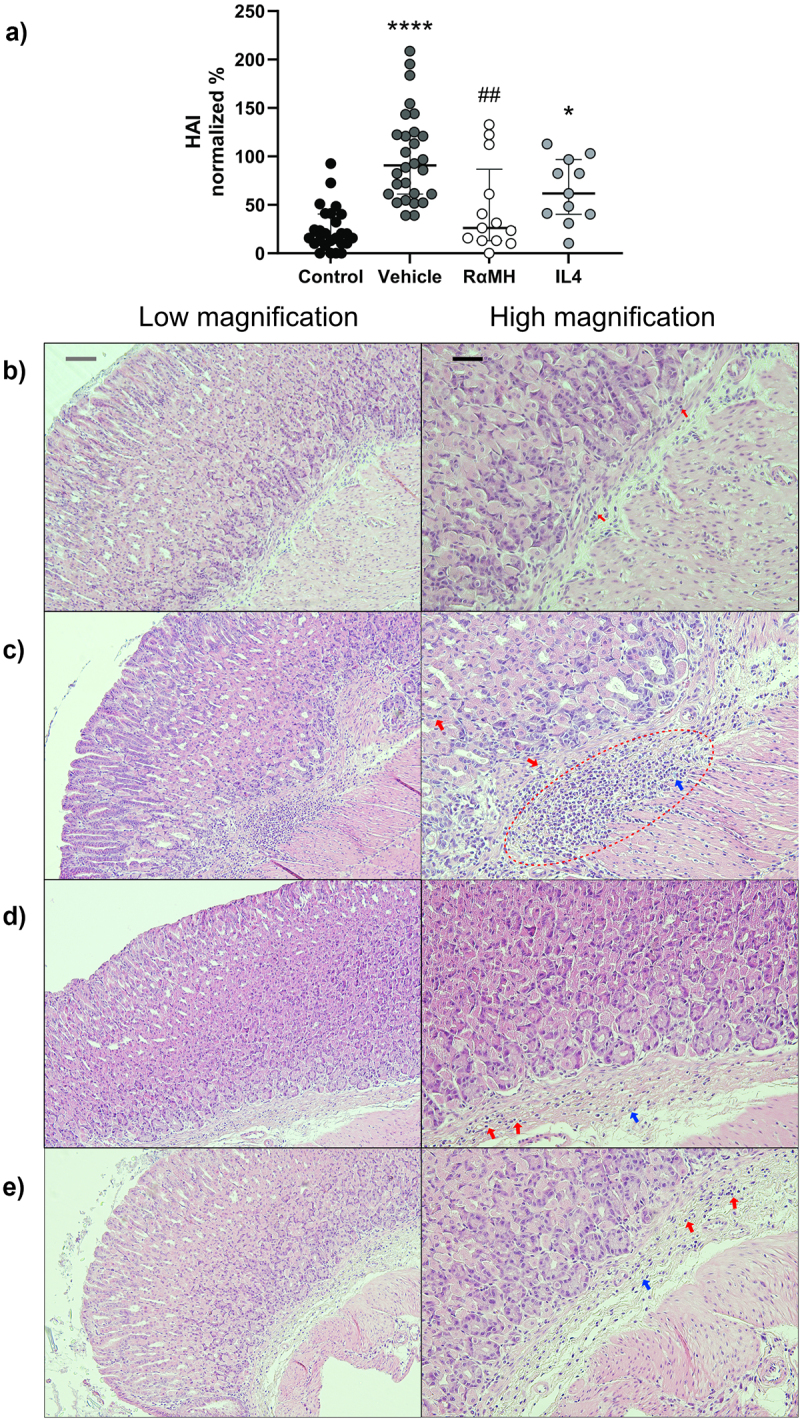
HAI in *H. pylori*-infected mice treated with RαMH and IL4. HAI was scored on H&E-stained tissue sections from non-infected control mice (Control, *n* = 26), *H. pylori*-infected vehicle-treated mice (Vehicle, *n* = 28), *H. pylori*-infected mice treated with RαMH (RαMH, *n* = 13) and *H. pylori*-infected mice treated with IL4 (IL4, *n* = 11). The HAI was classified according to the following criteria: inflammation, epithelial defects, oxyntic atrophy, hyperplasia, pseudo pyloric metaplasia, dysplasia, and mucus metaplasia, on a 1–4 scale [35]. (a) HAI scores 14 days post-infection (dpi). Results are presented in percentages normalized to *H. pylori*-infected vehicle-treated mice to allow for presenting several experiments in the same graph. Each circle represents the score of one mouse. The median HAI score for this group, pooled from all five experiments was 5.0. *p < 0.05, ****p < 0.0001 compared to non-infected controls; ##*p* > 0.01 compared to *H. pylori*-infected vehicle-treated mice; Kruskal–Wallis test by ranks followed by Dunn’s multiple comparison test, median ± IQR. (b-e) Representative H&E-stained tissue sections from the murine corpus. Grayscale bar 100 µm and black scale bar 50 µm. The red arrows mark lymphocytes, and the blue arrows mark mast cells. (b) Section from a non-infected control mouse. (c) Section from an *H. pylori*-infected vehicle-treated mouse. The dashed red elliptical highlights an aggregate of inflammatory cells (leucocytes). (d) Section from an *H. pylori*-infected mouse treated with RαMH. (e) *H. pylori*-infected mice treated with IL4.

As expected, the *H. pylori*-infected vehicle-treated mice presented with increased HAI compared to the non-infected mice (*p* < 0.0001, Figure 2a). Inflammation, characterized by the accumulation of leukocytes, both polymorphonuclear (PMN) and mononuclear cells, which was the aspect that contributed to the majority of the HAI score in all groups (Figures 2b–e and S2). This included sporadic inflammatory cells present in the non-infected control mice (Figure 2b). In infected groups, epithelial defects such as tattered epithelial cells, occasional dilated glands, and apoptotic and/or necrotic debris were observed. Infected vehicle-treated mice displayed a low level of oxyntic atrophy and more inflammatory cells compared to the non-infected mice (Figure 2c and Supplementary Figure S2). The inflammation was characterized by aggregates of lymphocytes, PMN cells, and mast cells in the submucosa and mucosa (Figure 2c). Administration of RαMH reduced HAI scores in infected mice (*p* < 0.01, Figure 2a,d) with a similar trend for IL4-treated mice (Figure 2a,e). Moreover, RαMH treated mice did not have HAI scores significantly higher than the non-infected control mice (Figure 2a). In mice treated with RαMH or IL4, no oxyntic atrophy was noted. The HAI scores were due to mild epithelial defects and low grade of inflammation characterized by the presence of lymphocytes, PMN cells, and mast cells in the submucosa (Figure 2d–e). The scores for the individual parameters making up the HAI are available in the SND Service (see Data Availability section).

### RαMH and IL4 treatment decreased the *H. pylori* density in the mouse stomach

**Figure 3.**
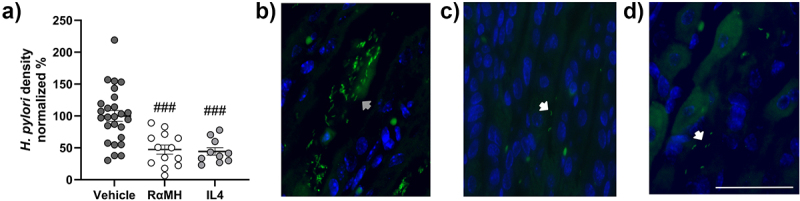
*H. pylori* density in infected mice with and without RαMH and IL4 treatment. *H. pylori* was visualized using a *Helicobacter* genus-specific fluorescent probe (green) on tissue sections from non-infected control mice (*n* = 26), *H. pylori*-infected vehicle-treated mice (Vehicle, *n* = 26), *H. pylori*-infected mice treated with RαMH (RαMH, *n* = 13), and *H. pylori*-infected mice treated with IL4 (IL4, *n* = 10). The bacteria in the surface mucus layer can be affected or lost during tissue harvesting and fixation. Hence, to avoid artifacts only *Helicobacter* in the gastric pits were counted. (a) Blinded visual semi-quantification of *H. pylori* density in mouse corpus pits 14 dpi. Results are pooled from five experiments and presented in percentage, normalized to *H. pylori*-infected vehicle-treated mice. Each circle represents one mouse. Statistics: ### *p* < 0.001 compared to *H. pylori*-infected vehicle-treated mice; one-way ANOVA, Tukey’s post hoc test, mean ± S.E.M. (b-e) Representative images of *Helicobacter* visualized in green by fluorescent *in situ* hybridization (FISH) and nucleus in blue (DAPI) in murine gastric tissue sections at 14 dpi, white scale bar 25 µm. (b) Image from an *H. pylori*-infected vehicle-treated mouse. The gray arrow marks a cluster of *H. pylori* in the gastric pits. (c) Image from an *H. pylori*-infected mouse treated with RαMH. (d) Image from an *H. pylori*-infected mouse treated with IL4. The white arrows mark isolated *H. pylori* in the gastric pits.

During harvesting and processing of the gastric tissue, much of the surface mucus layer is lost, as well as the bacteria residing in it. However, in areas where patches of mucus are still preserved, it is possible to detect commensal bacteria and *Helicobacter* (Supplementary Figure S3a–d). Due to the risk that the remaining surface mucus is not representative of the whole mucus layer, only *Helicobacter* in the gastric pits were quantified. No *Helicobacter* were found in the stomach of non-infected control mice. Some rod-shaped bacteria, likely lactobacilli [40], were present on the surface of the gastric mucosa in the areas where patches of mucus were still preserved (Supplementary Figure S3a). Treatment with RαMH or IL4 decreased the *H. pylori* density in the gastric pits by more than 50% compared to vehicle-treated mice (*p* < 0.0001, Figure 3a). In the *H. pylori*-infected vehicle-treated mice, the corpus pits were filled with many, often large, clusters of *H. pylori* (Figure 3b and Supplementary Figure S3b). In *H. pylori*-infected mice treated with RαMH or IL4, there were fewer *H. pylori* in the gastric pits (Figure 3c,d, respectively). Furthermore, the *H. pylori* in these treatment groups were spread in the gastric pits, either as single bacteria or occasionally in small clusters (Figures 3c,d and S3c,d).

### IL4 treatment increased the abundance of sialic acids on mucin O-glycans compared to control and RαMH treated groups

**Figure 4.**
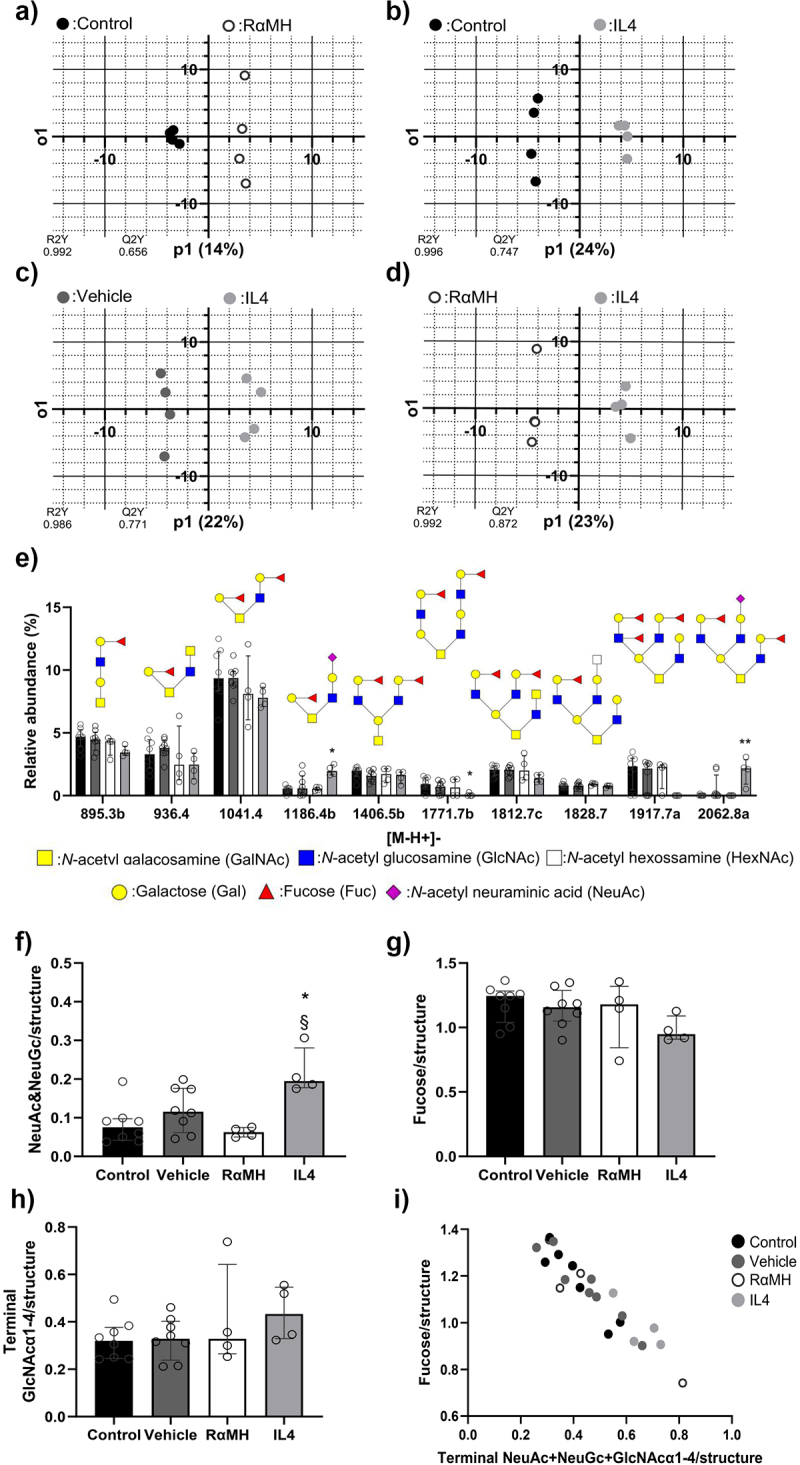
Corpus mucin O-glycosylation. O-glycan analysis using LC-MS/MS on non-infected control mice (Control, *n* = 8), *H. pylori-*infected vehicle-treated mice (Vehicle, *n* = 8), *H. pylori*-infected mice treated with RαMH (RαMH, *n* = 4) and *H. pylori*-infected mice treated with IL4 (IL4, *n* = 4). (a-d) OPLS-DA scores plots with p1 (predictive component) and o1 (orthogonal component). R2Y and Q2Y show actual and simulated models after random permutations of values i.e. prediction of training data and prediction of test data, respectively (*n* = 4). The closer to one the values are, the more reliable the model is. The percentage represents the variance explained by the predictive component. (e) The top 10 VIP glycans from the combined four OPLS models with an average relative abundance over 0.5%. *p < 0.05, ***p < 0.001 compared to non-infected controls. (f) The number of NeuAc and NeuGc/glycan structure in the groups. *p < 0.05 compared to control; § *p* < 0.05 compared to RαMH. (g) The number of fucose/glycan structure. (h) The number of terminal GlcNAcα1–4/structure. i) Plot of fucose/structure against terminal NeuAc, NeuGc and GlcNAcα1–4/structure. Statistics: Kruskal-Wallis H-test with Dunn´s multiple comparison and Spearman rank correlation. Bars represent median ± IQR.

Since mucin glycans regulate *Helicobacter* adhesion and growth, the effect of the treatments on glycan composition and structure was investigated using LC-MS [14]. A total of 91 corpus mucin O-glycans were identified among all groups. OPLS-DA was run to examine the most important O-glycans that differ between groups. Four OPLS-DA were able to make a model Control vs RαMH, Control vs IL4, Vehicle vs IL4 and RαMH vs IL4 (Figure 4a–d). The glycans that contributed to the differences between the groups (VIP) and had over 0.5% average relative abundance were further examined (Figure 4e). Many of the O-glycans were composed of core 2 glycans with a high degree of fucosylation. The median number of sialic acids (NeuAc and NeuGc) per glycan was an order of magnitude lower than for fucose. The lowest median number of sialic acids per glycan was found in the infected mice treated with RαMH (0.063 IQR:0.050–0.075) and non-infected control mice (0.075 IQR: 0.042–0.097), thereafter infected mice treated with vehicle (0.116 IQR:0.062–0.176) and IL4 (0.195 IQR:0.178–0.281). Kruskal–Wallis H-test analysis demonstrated a statistically significant increased median number of sialic acids per glycan structure among IL4-treated mice compared to the non-infected control (*p* < 0.05) and RαMH treated mice (*p* < 0.05, Figure 4f). The increased levels of terminal sialic acid and a trend to increase in terminal GlcNAcα1–4 containing O-glycans in the IL4 treated group could possibly explain why this group had a trend toward lower median number of fucose per structure since the sialic acids and GlcNAcα1–4 compete with fucose for many of the same precursor structures (Figure 4g,h). This was confirmed by plotting the average fucose/structure versus the average number of NeuAc, NeuGc, and terminal GlcNAcα1–4/structure. The monosaccharides were inversely proportional and correlated significantly (*p* < 0.0001) with a Spearman rank coefficient of −0.93 (Figure 4i).

### RαMH did not have a cytotoxic effect on H. pylori

**Figure 5.**
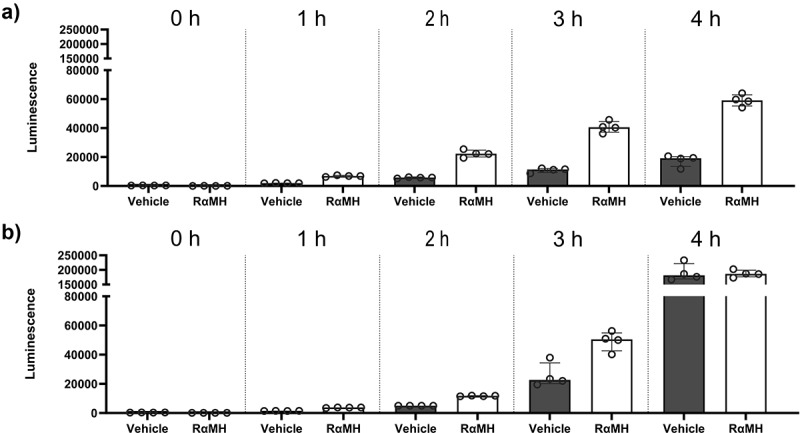
RαMH did not have a cytotoxic effect on *H. pylori* SS1. in the *in vivo* experiments, the infected mice received either PBS with 6.25% DMSO (vehicle) or RαMH in PBS with 6.25% DMSO (RαMH). To mimic this, vehicle (*n* = 4) and RαMH (*n* = 4) were added to *H. pylori* in brucella broth under microaerobic conditions, and cell growth/viability was measured as the reducing potential of viable cells (luminescence) for 4 hours. (a) *H. pylori* in PBS containing 6.25% DMSO (vehicle), *H. pylori* in PBS containing 12.5 mg/ml RαMH, and 6.25% DMSO (RαMH). (b) The same reagents were diluted 2.5 times in PBS to mimic the gastric concentration of the compounds. Bars represent median ± IQR. Kruskal–Wallis test by ranks followed by Dunn’s multiple comparisons. The experiment was performed a second time with similar results (See Supplementary Figure S4).

A cell viability assay was performed to assess if RαMH had a direct cytotoxic effect on *H. pylori*. The time frame that the gastric content is expected to pass from the stomach to the intestine is approximately 4 h [41]. In the *in vivo* experiments, the infected mice received either vehicle solution (PBS containing 6.25% DMSO) or RαMH in PBS containing 6.25% DMSO. When fed to mice by oral gavage, compounds are diluted with the gastric content. Hence, we also analyzed *H. pylori* growth in vehicle solution and RαMH diluted 2.5 times more than in the *in vivo* experiment. None of these experimental setups showed that RαMH inhibited *H. pylori* growth during the 4 h investigated (Figures 5 and S4).

### Neither RαMH nor IL4 treatment altered the expression of *H. pylori* virulence factors

**Figure 6.**
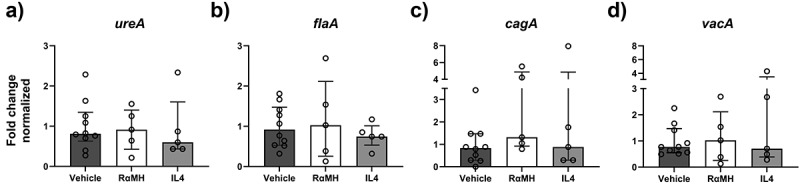
Expression of virulence factors in *H. pylori* from mice treated with vehicle, RαMH or IL4. (a-d) Expression of *H. pylori* virulence factors in stomachs of *H. pylori-*infected vehicle-treated mice (Vehicle, *n* = 10), *H. pylori*-infected mice treated with RαMH (RαMH, *n* = 5) and *H. pylori*-infected mice treated with IL4 (IL4, *n* = 4) analyzed by RT-PCR and normalized against *H. pylori 16s*. The results are expressed in Fold change and normalized to *H. pylori* from infected vehicle-treated mice. No significant differences between the different treatment groups were observed; Kruskal-Wallis H-test followed by Dunn’s multiple comparisons. Bar median ± IQR. (a) Urease subunit α, *urea*. (b) Flagellin A, *flaA*. (c) Cytotoxin-associated gene A, *cagA*. (d) Vacuolating cytotoxin A, *vacA*.

To investigate if the treatments induced alterations in *H. pylori* virulence, the expression of virulence factors were assessed by RT-PCR and transcript levels normalized against the *H. pylori 16s* reference gene. No differences in expression levels of urease subunit α (*urea*), flagellin A (*flaA*), cytotoxin-associated gene A (*cagA*) and vacuolating cytotoxin A (*vacA*) were detected between *H. pylori*-infected mice treated with vehicle, RαMH or IL4 (Figure 6a-d).

### Treatment with RαMH and IL4 did not affect serum anti-*H.* *pylori* IgG or antimicrobial peptide (AMP) expression levels, but IL4 treatment increased the expression of Trefoil factor family 1 (*Tff1*)

**Figure 7.**
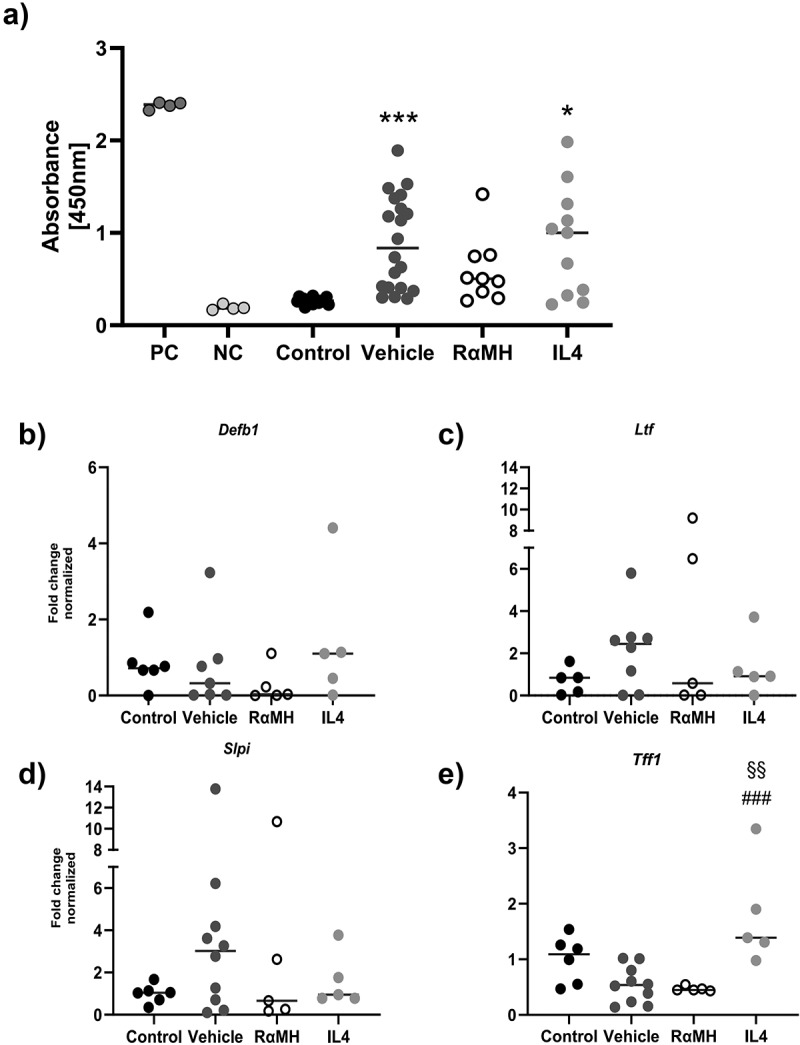
Anti-*H. pylori* IgG levels in serum and expression of peptides in mouse corpus. (a) Mouse serum IgG response to *H. pylori* was determined by ELISA at 14 dpi in non-infected control mice (control, *n* = 18), *H. pylori*-infected vehicle-treated mice (Vehicle, *n* = 20), *H. pylori*-infected mice treated with RαMH (RαMH, *n* = 9) and *H. pylori*-infected mice treated with IL4 (IL4, *n* = 11). Serum from an *H. pylori*-infected mouse 14-weeks pi was used as a positive control (PC, *n* = 1, 4 technical replicates) and serum from a germ-free mouse as a negative control (NC, *n* = 1, 4 technical replicates). Results are presented in absorbance at 450 nm. *p < 0.05, ***p < 0.001 compared to non-infected controls; Kruskal-Wallis H test followed by Dunn’s multiple comparison test, each dot represents one mouse, bars represent the median. There was no statistically significant difference between the vehicle-treated mice and mice treated with RαMH or IL4. (b-e) Gene expression in corpus from control (*n* = 5–6), vehicle (*n* = 7–10), RαMH (*n* = 4–5), and IL4 (*n* = 5) mice analyzed by RT-PCR. The results are expressed as Fold change and normalized to non-infected control mice using β actin as the housekeeping gene. The bars represent the median of each group. (b) Defensin β1, *Defb1*. Kruskal-Wallis H-test followed by Dunn’s multiple comparisons. (c) Lactoferrin, *Ltf*. One-way ANOVA, Tukey’s post hoc test. (d) Secretory leucocyte protease inhibitor, *Slpi*. Kruskal-Wallis H-test followed by Dunn’s multiple comparisons. (e) Trefoil factor 1, *Tff1*. One-way ANOVA, Tukey’s post hoc test. §§ *p* < 0.01 compared to RαMH, ### *p* < 0.001 compared to vehicle. The absence of a symbol indicated that no statistically significant difference was detected.

Serum anti-*H. pylori* IgG levels were used as a proxy for activation of the adaptive immune response [42]. Infected vehicle-treated mice had higher serum anti-*H. pylori* IgG levels than non-infected controls (*p* > 0.0001, Figure 7a). Infected mice treated with RαMH and IL4 had serum anti-*H. pylori* IgG levels similar to the vehicle-treated infected mice (Figure 7a). Furthermore, there were no significant differences in gene expression levels of the antimicrobial peptides: Defensin β 1 (*Defb1)*, Lactoferrin (*Ltf*), or Secretory Leukocyte Peptidase Inhibitor (*Slpi*) (Figure 7b-d). However, expression of the Tff1 (*Tff1*) peptide was increased in *H. pylori*-infected mice treated with IL4 compared to infected-vehicle and RαMH-treated mice (Figure 7e).

### Expressions of factors that regulate mucus production were affected by RαMH and IL4 treatment

**Figure 8.**
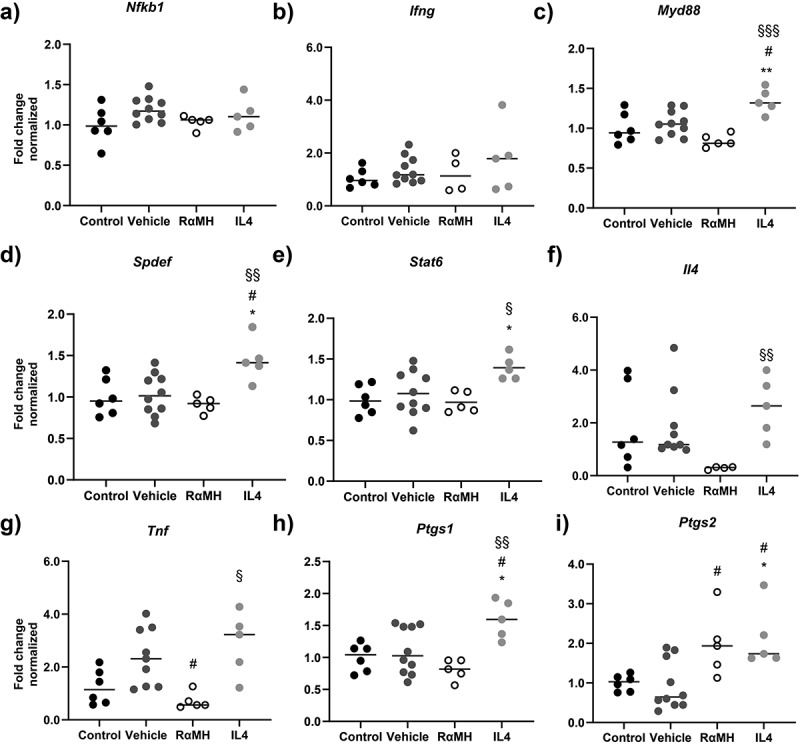
Expression of factors regulating mucus production in mouse corpus. (a-i) Gene expression in the corpus from non-infected control mice (Control, *n* = 6), *H. pylori*-infected vehicle-treated mice (Vehicle, *n* = 9–10), *H. pylori*-infected mice treated with RαMH (RαMH, *n* = 4–5) and *H. pylori*-infected mice treated with IL4 (IL4, *n* = 5) analyzed by RT-PCR. The results are normalized to non-infected control mice using β actin as the housekeeping gene and expressed as Fold change normalized to non-infected control mice. The bars represent the median of each group. (a) Nuclear factor κ B1, *Nfkb*. (b) Interferon γ, *Ifng*. (c) Myeloid differentiation primary response gene 88, *Myd88*. (d) SAM pointed domain-containing Ets transcription factor, *Spdef*. (e) Signal transducer and activator of transcription 6, *Stat6*. Statistics: one-way ANOVA, Tukey’s post hoc test. (f) Interleukin-4, *Il4*.; Kruskal-Wallis H-test followed by Dunn’s multiple comparisons. (g) Tumor necrosis factor α, *Tnf*. (h) Prostaglandin-endoperoxide synthase 1, *Ptgs1*. (i) Prostaglandin-endoperoxide synthase 2, *Ptgs2*. Statistics: One-way ANOVA, Tukey’s post hoc test. *p < 0.05, **p < 0.01 compared to control; # *p* < 0.05 compared to vehicle; § *p* < 0.05, §§ *p* < 0.01, §§§ *p* < 0.001 compared to RαMH. No symbol indicates that no statistically significant differences were observed.

Finally, we investigated expression levels of factors previously shown to be important for the regulation of mucus production [21,43,44]. No statistically significant effects on the expression levels of nuclear factor κ B1 (*Nfkb1*) or Interferon γ (*Ifng)* were detected across the groups (Figure 8a,b). In mice treated with IL4, expression of myeloid differentiation primary response gene 88 (*Myd88*) and SAM pointed domain-containing Ets transcription factor (*Spdef)* was upregulated compared to all other groups (Figure 8c,d), and signal transducer and activator of transcription 6 (*Stat6*) was upregulated compared to non-infected controls and *H. pylori*-infected RαMH-treated mice (Figure 8e). IL4 treatment also increased the expression levels of IL4 (*Il4*, Figure 8f) and tumor necrosis factor α (*Tnf*, Figure 8g) compared to RαMH-treated mice. However, treatment with RαMH downregulated *Tnf* compared to the infected vehicle-treated mice (Figure 8g). Only IL4 treatment had a significant effect on prostaglandin-endoperoxide synthetase 1 (*Ptgs1*, Figure 8h) but both RαMH and IL4 increased the expression of prostaglandin-endoperoxide synthetase 2 (*Pgts2*, Figure 8i). Thus, several factors/pathways involved in mucus regulation were affected by the treatments and both cytokine environment and pathways indicated in the restoration of mucus production differed between the RαMH and IL4 treated groups.

## Discussion

In this study, we showed that RαMH and IL4 increased mucin production and transport in route secretion in gastric surface mucous cells and decreased *H. pylori* density during *H. pylori* infection. Treatment with RαMH and IL4 did not affect serum anti-*H. pylori* IgG, expression of antimicrobial peptides or *H. pylori* virulence factors and RαMH did not have a cytotoxic effect on *H. pylori*. Thus, it is probable that the decreased *H. pylori* density was due to the restored mucus production. The expression of cytokines and factors that affect mucus production as well as mucin O-glycan sialylation levels differed between mice treated with RαMH and IL4. Thus, increased mucus production via two different mechanisms can have similar effects on pathogen density in spite of differences in the local niche.

For our investigation, we used male C57BL/6 mice because they have a higher level of *H. pylori* colonization than females during early infection [45]. In the murine stomach, alterations caused by *H. pylori* infection are primarily located in the corpus [35]. Hence, the corpus of the stomach was the main region examined. After harvesting and fixation of gastric tissue, most of the surface mucus, *H. pylori* and *H. suis* residing in it are lost; however, the bacteria in the pits are not disrupted [14]. Additionally, *H. pylori* shows a preference to bind to highly differentiated pit cells found in the upper part of the gastric pit regions, where it translocates its virulence factors [46]. Therefore, *H. pylori* density was scored in the upper part of the pit region.

The mouse-adapted *H. pylori* SS1 strain used in this study, like other *Helicobacter* strains, has several virulence factors that contribute to the colonization of the gastric mucosa [47]. As previously shown with clinical *H. pylori* isolates differences in the expression of the bacteria’s virulence factors can impact *H. pylori* density and colonization capacity in mice [48]. To investigate if changes in expression of virulence factors were induced by RαMH or IL4, we analyzed the expression levels of a group of virulence factors, selected based on their contributions to several steps in the colonization process. *H. pylori* produces urease, an enzyme responsible for reducing gastric acidity and uses flagella for movement, allowing the bacteria to enter and colonize the mucus layer and migrate toward the epithelial cells. In the gastric environment, secreted toxins can cause cell damage in the host [1]. The vacuolating cytotoxin A (VacA) is a toxin that causes vacuolization, pore formation and apoptosis in the target cells [49]. The cytotoxin-associated gene A (*cagA)* is encoded in the cag-pathogenicity island (cagPAI) and it codes for the production of both CagA (an oncoprotein with cytotoxic effect on the host cell) and a type IV secretion system (T4SS) [4]. In *H. pylori* SS1 the cagPAI-harbored T4SS is non-functional partially due to the presence of a defective CagY protein but the strain is still classified as CagA-positive [47,50,51]. All primers were designed using *H. pylori* SS1. We confirmed that the expression of the virulence factors analyzed (*ureA, flaA, cagA* and *vacA*) were not altered by the treatments administrated to the mice.

Anti-*H. pylori* IgG antibodies can be detected in the murine serum of infected mice at 7 dpi [42]. We analyzed the antibody response at 14 dpi and, as expected, with infection there was an increase in the levels of anti-*H. pylori* antibodies. However, there was no increase in the levels of anti-*H. pylori* antibodies in the treatment groups. Furthermore, there were no increase in expression levels of AMPs or of glycans that decreased *Helicobacter spp* growth [11,14], and RαMH did not have a cytotoxic effect on *H. pylori*. The lack of effect on these parameters lends support to the notion that the decreased levels of *H. pylori* in the gastric mucosa were aided by the restored mucus production. Several factors can contribute to the beneficial effects of restoring mucus production on pathogen density; mucins can bind and remove *H. pylori* from the niche close to the epithelial surface [19,20,52]. Mucin O-glycans also inhibit quorum sensing and acquisition of antimicrobial resistance through natural transformation, which may render microorganisms less pathogenic [53,54]. Furthermore, defensive factors secreted into a strategic position in the mucus, such as antimicrobial peptides and IgA, likely lose their efficiency if the mucus layer is scant/dysfunctional [12]. AMPs are produced by the epithelial cells of the gastrointestinal tract and defend the mucosa against bacteria, viruses, and fungi [12]. Several AMPs have been shown to be affected by *H. pylori* infection: Defensin β 1 (*Defb1*) is upregulated 1 month after *H. pylori* infection in mice [55], and Lactoferrin (*Ltf*) is upregulated in *H. pylori*-infected patients [56], whereas Secretory Leukocyte Peptidase Inhibitor (*Slpi*) is decreased in *H. pylori*-infected patients [57,58]. We found no significant differences in the expression of these AMPs, which might be explained by the shorter duration of the infection in the current study and by that the inter-individual variation in the expression levels of *Ltf* and *Slpi* was relatively large in spite of Ct values being in an acceptable range (see methods). We found that mice treated with IL4 expressed higher levels of *Tff1* compared to both infected vehicle-treated and RαMH-treated mice. TFF peptides have gastroprotective roles, enhancing cell migration and promoting healing in mucous epithelia [59]. Moreover, TFF1 and MUC5AC are mainly secreted together by surface mucous cells, and TFF1 has been described as having a role in the intracellular assembly of MUC5AC [59,60]. Furthermore, TFF1 has been shown to bind *H. pylori* and slow *H. pylori* movement in mucus [61]. Thus, TFF1 may contribute to decreased *H. pylori* density in the murine stomach both by direct action on *H. pylori* and by contributing to the restored mucus production.

Mucins are continuously secreted from the gastric epithelial cells and confer the majority of their actions after they have been secreted [15]. Methods that quantify the amount of a protein in the tissue can therefore result in unclear results as production and secretion rate are important parameters in this context: a decreased secretion could lead to an increased amount of mucin in the cells, and conversely, an increased secretion could lead to a decreased amount of mucin in the cells, without a change in mucus production. Due to the large size of the mucin genes, the cell operates with multiple chaperons, prolonging the life of the transcripts and hence reducing transcription costs [62]. Additionally, a large part of the mucin molecule comprises glycans and is therefore subject to major post-translational regulation, and mucin mRNA levels often do not reflect mucin production or mucus thickness [21,22]. Therefore, metabolic labeling methods likely better reflect mucin production. Here we have used GalNAz, which labels the highly abundant mucin O-glycans (but not N-glycans) as a metabolic label [34,63].

Mice infected with *H. pylori* have previously been shown to have lower mucin production in the stomach than non-infected mice [15]. Likewise, we observed that *H. pylori*-infected vehicle-treated mice had a lower rate of mucin production than the non-infected mice in the current set of experiments. In mice treated with RαMH and IL4, we noted higher GalNAz label incorporation in corpus mucous cells, indicating an increase in the production of newly synthesized mucins and this increase restored the mucin production to levels similar to those of non-infected mice.

RαMH increases PGE_2_ production and acid production in mouse stomachs [27]. The thickness of the adherent gastric mucus layer has previously been shown to be increased in rats treated with RαMH after ethanol exposure, but not after RαMH treatment alone [23], suggesting that the increase in mucus production requires the presence of some form of stressor. Both PTGS1 and PTGS2 (also known as COX-1 and COX-2, respectively) can increase the levels of PGE_2_ by catalyzing the production of the precursor PGH_2_ [44]. PGE_2_ induces mucus secretion [44,64], and the increased mucus production we identified in response to RαMH-treatment in the current study is thus likely governed by PGE_2_ via *Ptgs2*.

*H. pylori* infection in humans, primates, and mice elicits a Th1/17 immune response characterized by the recruitment of primary T lymphocytes expressing IFN-γ to the stomach and reduced levels of type 2 T helper lymphocytes expressing IL4 [29,65–67]. IFN-γ-null mice do not exhibit an inflammatory response even in a chronic *H. pylori* infection setting. However, mice deficient in IL4 present a skewed type 1 T helper lymphocyte immune response to *H. pylori* infection [30]. In the *C. rodentium-*infection model, we have observed that IL4 treatment enhanced mucus production in colonic cells via the stat6/spdef pathway [21]. We also found higher levels of *Stat6* and *Spdef* in *H. pylori*-infected mice treated with IL4. Thus, it is likely that also in the *H. pylori* infection model used in the current study, the restored mucin production induced by IL4 treatment occurs partially via the stat6-spedf pathway, although additional factors involved in mucus production (including *MyD88*, *Ptgs1,* and *Ptgs2*) were also upregulated, suggesting that several pathways contributed to the restoration of mucus production. Our experiments demonstrated that IL4 has a gastroprotective effect on the mucosa by decreasing *H. pylori*-associated lesions and decreased the *H. pylori* density in the gastric pits. In line with these results, a previous study showed that IL4 prevented gastritis and lowered the pathogen density in *H. felis*-infected mice [30]. Thus, IL4 treatments decrease pathogen density in at least three infection models, likely via the same mechanism.

In the current study, *Il4*, *Tnf, Spdef,* and *Stat6* expression were increased in *H. pylori*-infected mice treated with IL4 but trended toward a decrease or no changes in mice treated with RαMH, further supporting that the mechanisms behind the increased mucin production of RαMH and IL4 treatment occur via different mechanisms. Future studies investigating the molecular pathways regulating mucus production of the gastric epithelial cell in detail would be beneficial.

In spite of more than 35 years of attempts to develop a vaccine against *H. pylori* infection, there are still no vaccines available. Several vaccine candidates induce specific antibody production and decrease the *H. pylori* density in the stomach [68]. Possibly, a way forward could be to combine treatments that have effect but do not succeed in eradicating *H. pylori*, for example by combining mucus-stimulating treatments with therapeutic vaccination.

In conclusion, both RαMH and IL4 treatment restored mucin production albeit via different mechanisms, and this restoration was accompanied by a decrease in *H. pylori* density. We cannot exclude that additional factors may contribute to the decrease in pathogen density; however, the fact that restoration of mucin production via two different means, together with that mucin bind and disseminate *H. pylori* and mice lacking the Muc1 or Muc5AC mucins have higher *H. pylori* densities after infection than wild-type mice [18–20,52], support that restoration of mucin production contributes to removing *H. pylori* from its gastric niche. The treatments with RαMH or IL4 also had a gastroprotective action but did not affect the host antibody titers, expression of AMPs or expression of *H. pylori* virulence factors. Our results suggest that restoring mucin production after *H. pylori* infection could be a viable approach to target the infection, possibly in combination with other therapies or different treatment regimens to eradicate *H. pylori.*

## Supplementary Material

Figure S4.jpg

Figure S2.jpg

Figure S3.jpg

Figure S1.jpg

Author Checklist Full checked_manus rev v2.pdf

## Data Availability

The MS data that support the findings of this study are openly available in GlycoPost (https://glycopost.glycosmos.org/) at https://glycopost.glycosmos.org/entry/GPST000464, reference number GPST000464. The data from the other analyses can be found openly available in the Swedish National Data (SND) Service (https://snd.se/en) at https://doi.org/10.5878/hg1a-sd78.
