# Supplementary figures and images for: Treatment with (R)-α-methylhistamine or IL4 stimulates mucin production and decreases Helicobacter pylori density in the murine stomach
Source: Virulence. 2025 Jul 16;16(1):2530173. doi: 10.1080/21505594.2025.2530173 (PMC12269685; doi:10.1080/21505594.2025.2530173)

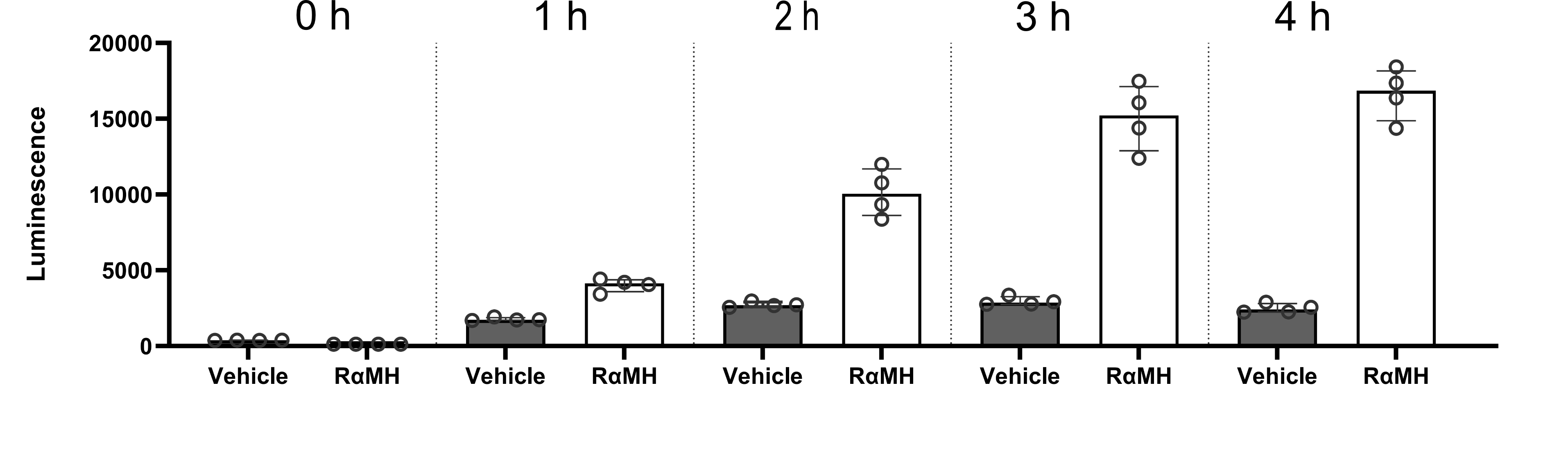

Supplement: Figure S4.jpg [file KVIR_A_2530173_SM7117.jpg]

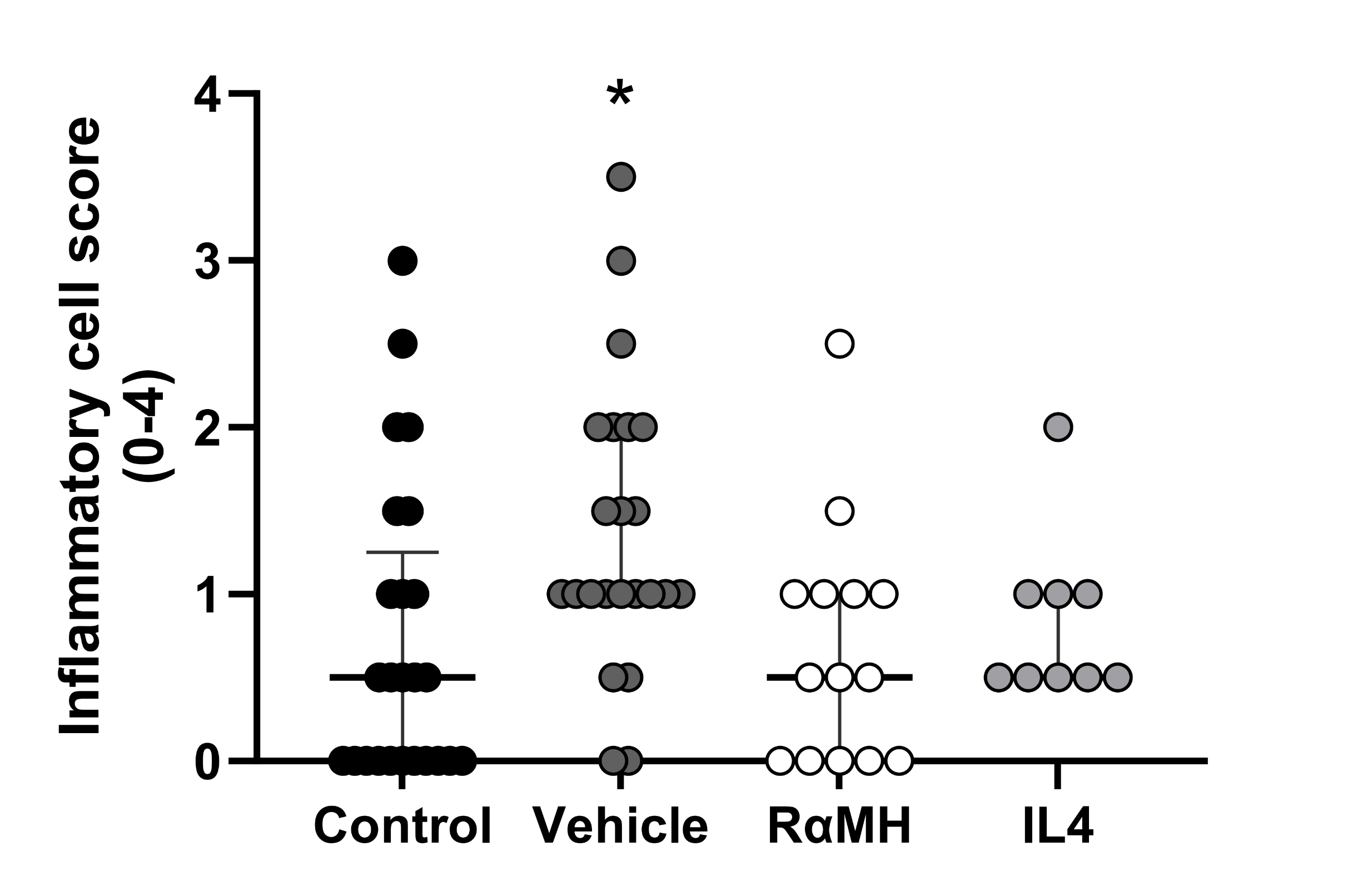

Supplement: Figure S2.jpg [file KVIR_A_2530173_SM7116.jpg]

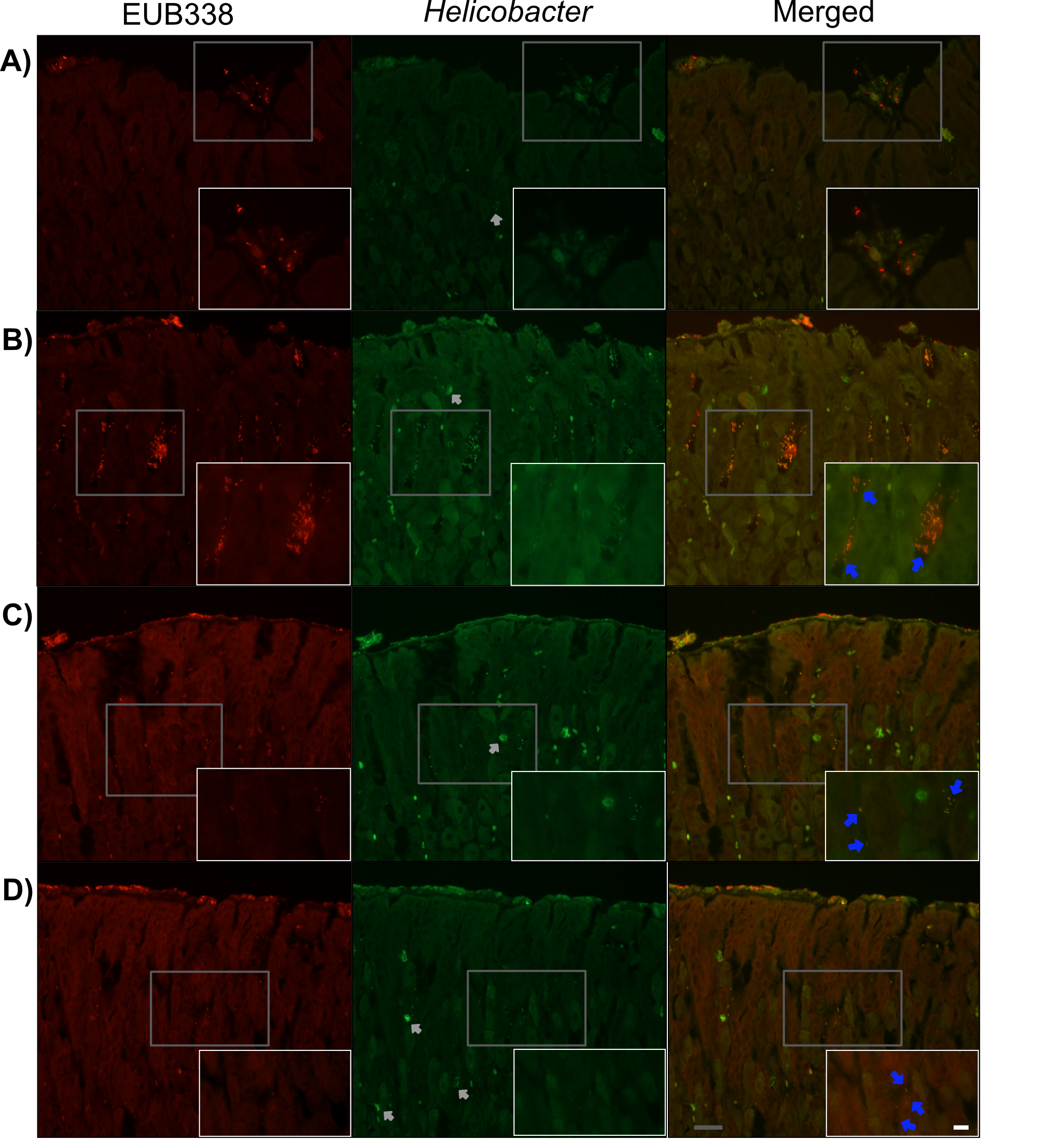

Supplement: Figure S3.jpg [file KVIR_A_2530173_SM7115.jpg]

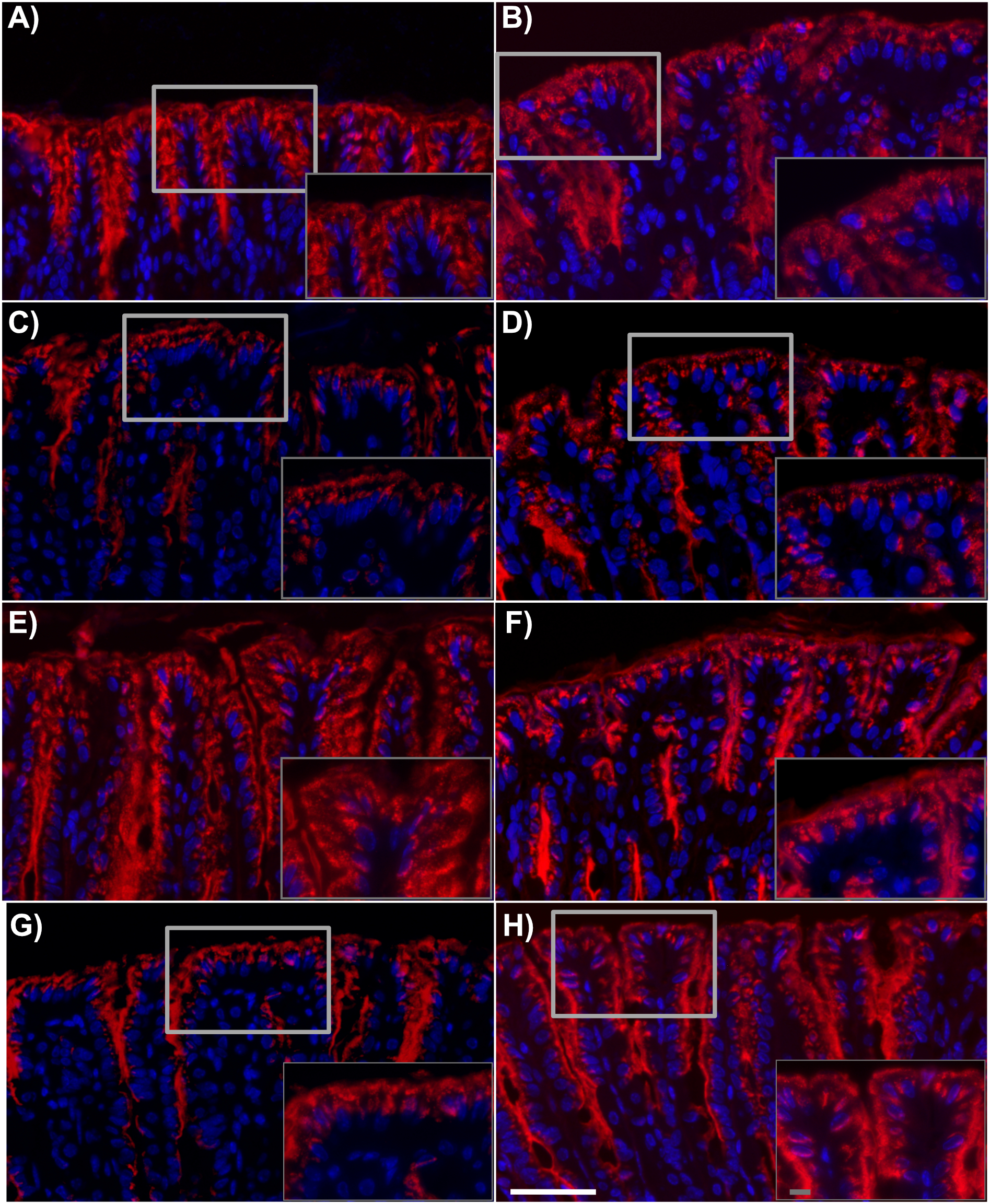

Supplement: Figure S1.jpg [file KVIR_A_2530173_SM7114.jpg]
